# Cliophysics: Socio-Political Reliability Theory, Polity Duration and African Political (In)stabilities

**DOI:** 10.1371/journal.pone.0015169

**Published:** 2010-12-29

**Authors:** Alhaji Cherif, Kamal Barley

**Affiliations:** 1 Mathematical Institute, University of Oxford, Oxford, United Kingdom; 2 School of Human Evolution and Social Change, Arizona State University, Tempe, Arizona, United States of America; Universita' del Piemonte Orientale, Italy

## Abstract

Quantification of historical sociological processes have recently gained attention among theoreticians in the effort of providing a solid theoretical understanding of the behaviors and regularities present in socio-political dynamics. Here we present a reliability theory of polity processes with emphases on individual political dynamics of African countries. We found that the structural properties of polity failure rates successfully capture the risk of political vulnerability and instabilities in which 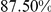, 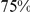, 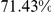, and 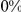 of the countries with monotonically increasing, unimodal, U-shaped and monotonically decreasing polity failure rates, respectively, have high level of state fragility indices. The quasi-U-shape relationship between average polity duration and regime types corroborates historical precedents and explains the stability of the autocracies and democracies.

## Introduction

Beginning with the Berlin Conference in 1884 – leading to the colonial despotism, to the National Conference of 1989 in Benin – marking the beginning of the democratic renewal, Africa continues to experience various patterns of political-economic regulations, mostly shifting away from autocratic regimes and toward more open governance. The continent has seen an increasing number of incomplete transitions to democracy, resulting in a high vulnerability to outbreaks of conflict [1]–[6]. Hypotheses for the proneness of the continent to conflicts include the inherent contradictions of being both partly open and repressive, the political incoherencies associated with anocratic (semi-democratic) regimes, economic underdevelopment, poor managerial styles, and ethnic politics and polarization [7]–[15]. However, in the past decades, there has been fewer political instabilities in anocratic regimes than predicted by historical data and previous studies. Here we develop a simple model of polity duration that explains historical data, and shows that the risk of polity change depends on the structural properties of polity failure rates. Three cliophysical parameters [16] quantifying the properties of the risk of political vulnerabilities are estimated from the data, and are used to provide a scientific understanding of the underlying mechanisms of regime stability. We call this socio-political reliability theory, and it has relevant implications for policy evaluations and designs.

Political processes within Africa have been characterized by waves of political instabilities, economic under-performances and other social factors with more than half of the sub-Saharan countries having experienced some forms of state-formation and/or post-independence civil conflicts since 1960, making the continent a testbed for exploring the determinants of political instabilities and state failures. As a result, we have seen an upsurge of literature addressing the causes of civil wars and political unrests. Understanding the causes and consequences of these civil conflicts have been a focus for most of the recent social science research, in particular sociological, political and economic inquiries with in-depth studies highlighting the role of economic under-development and economic grievances in shaping risks of conflicts as illustrated by the works of Auvinen [1], Hauge and Ellingsen [12], and Collier et al. [5]; of monarchical tendency, oppression of minority groups and repressive managerial styles of african leaders as exemplified by the works of Francisco [9], Lichbach [17], and Moore [18]; of the ethno-linguistic and religious diversity, ethnic fractionalization, political polarization, favoritism and nepotism as identified by the works of Ellingsen [7], Horowitz [15], Gurr [10], Lichbach [19] and Vanhanen [20]; and comparatively, of the historical precedents in the works of Bratton and van de Walle [2], Ellingsen and Gleditsch [8], Hibbs [14], Huntington [6], and Muller and Weede [21]. Some of these scholarly works have provided insights into the subtleties of socio-political processes underlying the historical precedents and constraints affecting the effectiveness of Africa's political behaviors, and have resulted in accumulation of empirical data and studies. Despite these accumulations of empirical knowledge, theoretical contributions linking the polity dynamics, risk of political instabilities, and scientific understanding of the processes have been modest. In this paper, we provide quantitative evidence linking the structural properties of polity failure rates to variations of political dynamics, finding substantial supports and correlations between state fragility indices and the functional shape of polity failure rates.

## Materials and Methods

The model described herein links the levels of democracy, polity durations and properties associated with the risk of regime failure. To quantify the relationship between the polity duration and regime type, we use the polity score from the Polity IV data set [22], a scaled metric that measures regime governance and the extent to which countries are democratic. The score ranges start from 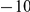 for fully autocratic to 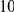 for fully democratic, and was originally developed by Gurr and colleagues [22], [23]. For our purpose, we map the polity score from 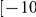, 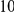] to 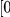, 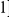. In this paper, we use polity duration as a mean of assessing and measuring sociopolitical reliability, dynamics of which are affected by various micro- and macro-sociopolitical and economic processes (e.g., managerial style of the leaders, protests and repressive measures to prevent riots). Possible processes that might affect polity dynamics are the changes in the governance and/or the structures of the government. This could be peaceful or rebellious transition (e.g., coup d' états). Another example in which polity dynamics can change is in the managerial styles. That is, the adaptation of a larger policy changes and international pressures can influence the dynamics of polity. Governments' attempts to minimize its probability of collapse or to maintain law and order can result in movements towards a repressive or an expansionary economic measure, and/or political repression or liberation. This in turn leads to changes in the level of democracy on the autocratic- democratic regime spectrum, and forces the state to move towards harshly autocratic or open governance. Polity change serves as an aggregative dynamical quantity that can provide some insights into the macro-sociopolitical behaviors originating from the complex interactions between the economic, social and political dynamics and domains. As a result analyses presented herein focus on the dynamics of polity and its duration.

**Figure 1:**
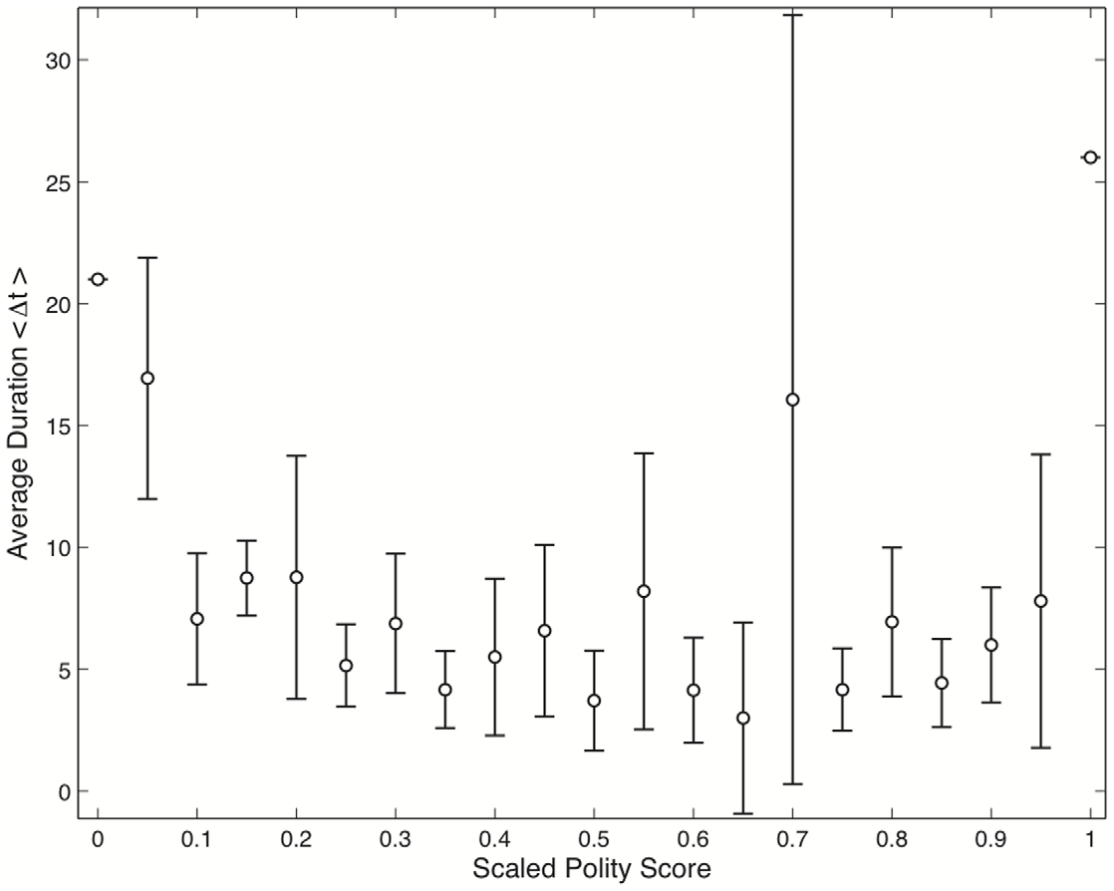
Level of democracy and polity durations. The figure shows the average polity duration, 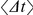 (years), with polities at either ends having longer duration.

Aggregating polity levels, we found that the average polity durations for highly autocratic and institutionally consistent democratic regimes are longer than the anocratic ones (e.g., see Table 1), in which autocracies (e.g., scaled polity score range 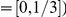 are estimated to be slightly longer than democracies in statistically insignificant way. However, fully or harshly autocracies (e.g., scaled polity score  = 0) have shorter average polity duration than fully democracies (e.g., scaled polity score  = 1). Table 1 summarizes our results. Figure 1 shows an average duration as a function of scaled polity score, in which anocratic regimes are highly unstable with majority of the polities experiencing some regime change within the first five years. The regime volatility is inversely proportional to the average polity duration (e.g., 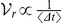). That is, the shorter the polity duration, the higher the political volatility and/or vulnerability to regime de-consolidation. In other words, countries that experience higher fluctuations in the level of polity in a short time have more vulnerable to political instabilities and civil unrests. Our findings corroborate this claim and the fact that both fully autocratic and democratic regimes are more durable and stable than anocratic regimes, and have been supported by numerous political scientists [17], [19], [20], [24]–[28]. However, we observed that anocratic regimes have relatively experienced a fewer civil unrests leading to political instabilities in the past decades than suggested by Table 1 and fig. 1. To study the mechanisms that explain this, we look at the cliometric space diagram of the shape parameters characterizing these polity failure rates to show that the structural properties of the polity failure rates capture the risks of political instabilities.

**Table 1:** Empirical justification of average polity duration for different regime type from 1946-2008. rom the transformed polity score, autocracy is a polity with a score in the range [0, 1/3], anocracy within the range [1/3, 2/3], and democracy within the range [2/3, 1].

Regime Type	Mean Polity duration, 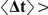	95% CI	N
Autocracies	10.65	(9.75, 11.56)	164
Anocracies	5.04	(5.02, 5.07)	53
Democracies	10.20	(8.42, 11.99)	78

To explore the direct role of functional shape of polity failure rate in explaining the historical risk of conflict and political instabilities, we use a modified Weibull function with a dispersive scale parameter, 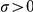, and *cliometric* shape parameters 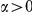 and 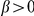 to characterize the distribution of polity duration, 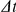:
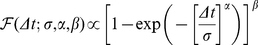
(1)


where the dispersive scale parameter 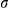 is the characteristic reference duration and is related to the mean polity duration, and shape parameters 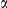 and 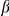 measure the susceptibility of the polity to socio-political conditions. We use proportionality to indicate that some of the countries do not perfectly follow the power-law breakdown rule we have assumed in order to arrive at Eq. 1 under a non-constant risk of polity change. The exponentiated form of Weibull function is used due to its flexibility to assume different structural forms with broader class of monotone hazard rate, and it is computationally convenient. It also contains distributions with non-monotonic structures (see [29] and references therein, and Appendix A). For instance, depending on the values of the shape parameters, we can approximate exponential, symmetric and asymmetric sigmoidal shapes, step function. Its flexibility with practical relevance and applicability have been demonstrated in various studies in reliability theory, composite materials, and bio-demography. Furthermore, from practical and comparative viewpoints, Weibull approximation and its exponentiation easily provide us a direct mean of connecting different layers of analysis from individual countries and regional groupings to the continent.

**Figure 2:**
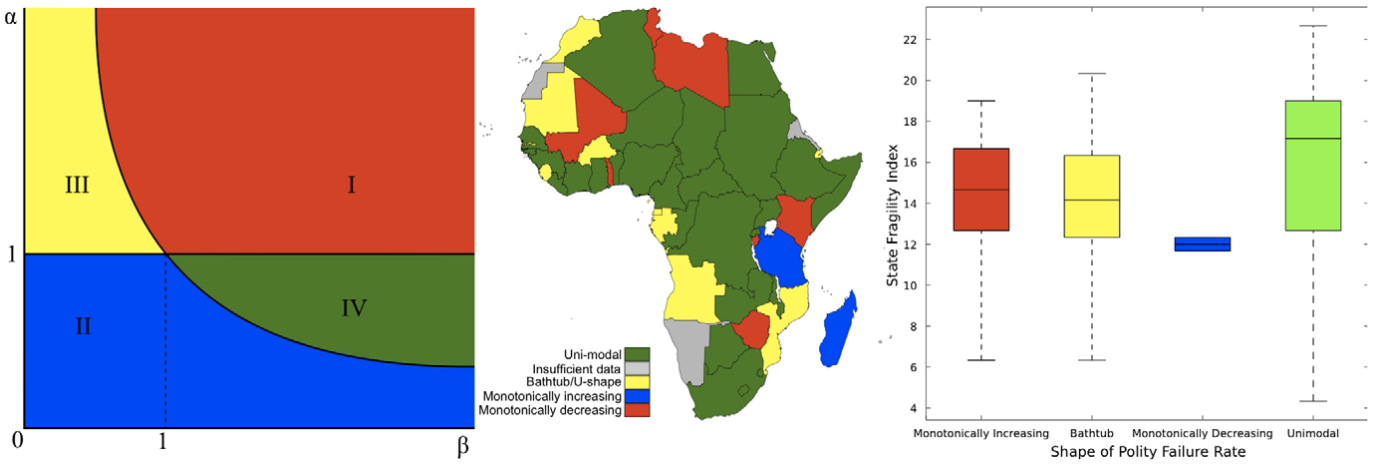
Properties of the polity failure rate or political hazard. The figures show: (a) functional type defining the risk of polity change (failure or hazard rate) in 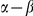 plane separated by the curves 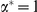 and 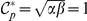; and (b) corresponding countries in Africa, as estimated by our model using least absolute regression with Trust-Region and Levenberg-Marquardt algorithms whenever possible. Interestingly, 87.50%, 0%, 71.43%, and 75% of African countries in regions I, II, III and IV, respectively, have extremely high state fragility indexes (c).

We employ a standard analytic procedures for lifetime data for estimating the parameters. Fitting our model to Polity IV data set for forty-eight African countries that have sufficient data points for our estimation problem [22], [23], [29], [30], we then characterize the risk of polity change by four distinct regions (e.g., see fig. 2 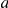) in the cliometric diagram of shape parameters 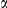 and 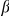 over which the failure rate as a function polity duration is, respectively, increasing monotonically (region I, e.g., 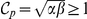 and 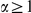), decreasing monotonically (region II, e.g., 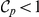 and 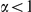), U-shaped or bathtub-shaped (region III, e.g., 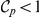 and 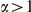), and unimodal (region IV, e.g., 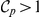 and 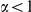). The Cliometric Number, 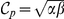 is defined here as the geometric mean of the two susceptibility (shape) parameters, and measures the initial growth of risk at the early stage of a new polity establishment. 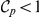 indicates an initial decay in the risk of polity change, while 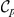 provides an indication of a possible increase in the vulnerability of the polity. The cliometric shape parameter 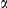 determines the monotonicity. That is, the polity failure rate is monotone (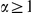, 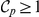 or 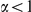, 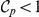) or non-monotone (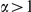, 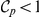 or 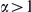, 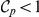) if the direction of both 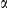 and 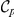 are the same or opposite, respectively. The conditions above are based on the derivative of the failure rate, as defined by 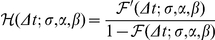. That is, the analysis of 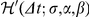 or equivalently of the transformation 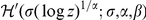, where 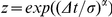 determines the four distinct regions. Figure 2 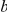 show our empirical summary for African continent. Interestingly, we found that the majority of African countries have either U-shape (22% or 11 countries) or uni-modal (56% or 27 countries) polity failure rates.

## Results and Discussion

U-shaped polity failure rate suggests that there is an initial period of high level of uncertainty and civil unrest in a newly formed polity, followed by a long or short constant failure rate as dissidents are abandoned or integrated into the political system, and/or the government satisfies the political demands of the dissidents. However, as the loss of political legitimacy increases, the state effectiveness decreases, and the lifetime of the polity is prolonged, the risk of polity change is heightened due to cumulative risks. Depending on the regime type, especially for anocracies, this heightening of risk of polity change can easily lead to political instability and civil unrest. For autocracies, this implies the strengthening of repressive measures and institutions as the regimes attempt to reduce the risks of their failures. U-shaped polity failure rate is reminiscent of three fundamental mechanisms driving the risk of polity change, namely the quality of governance, socio-political and economic factors, and management styles. We found that more than seventy percent (71.43%) of the countries with bathtub-shape failure rates have extreme to serious level of state fragility index (e.g., see Fig. 2*c*), a measure of state effectiveness and legitimacy in the areas of security, economics, development, and governance [22].

**Figure 3:**
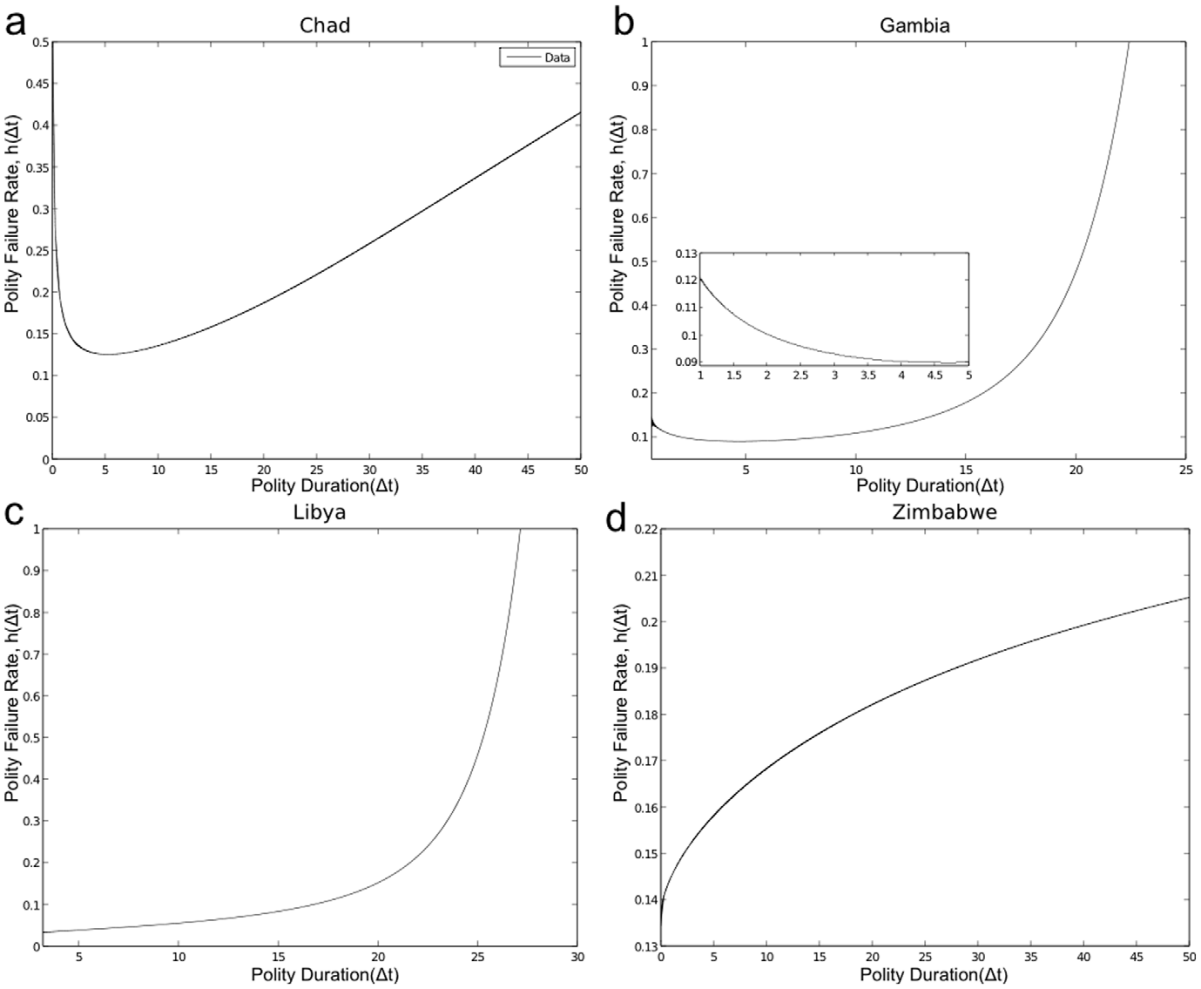
Polity Failure Rates. The figures show different properties for polity failure rates using estimated parameters and data sampled from eight different countries: (a) Chad with 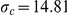, 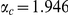, 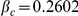; (b) Gambia with 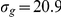, 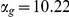, 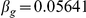; (c) Libya with 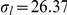, 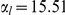, 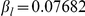; (d) Zimbabwe with 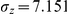, 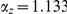, 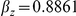, respectively. These parameters were estimated from empirical data using Least Absolute Regression method with Trust-Region and Levenberg-Marquardth algorithms, and were used to generate the polity failure rates: Chad and Gambia have U-shaped with Chad having a shorter constant failure rate and Gambia with shorter early life and longer constant polity failure rate before the increasing cumulative damage effect stage sets take over; Libya and Zimbabwe exhibit, respectively, convex and concave monotonically increasing failure rates.

**Figure 4:**
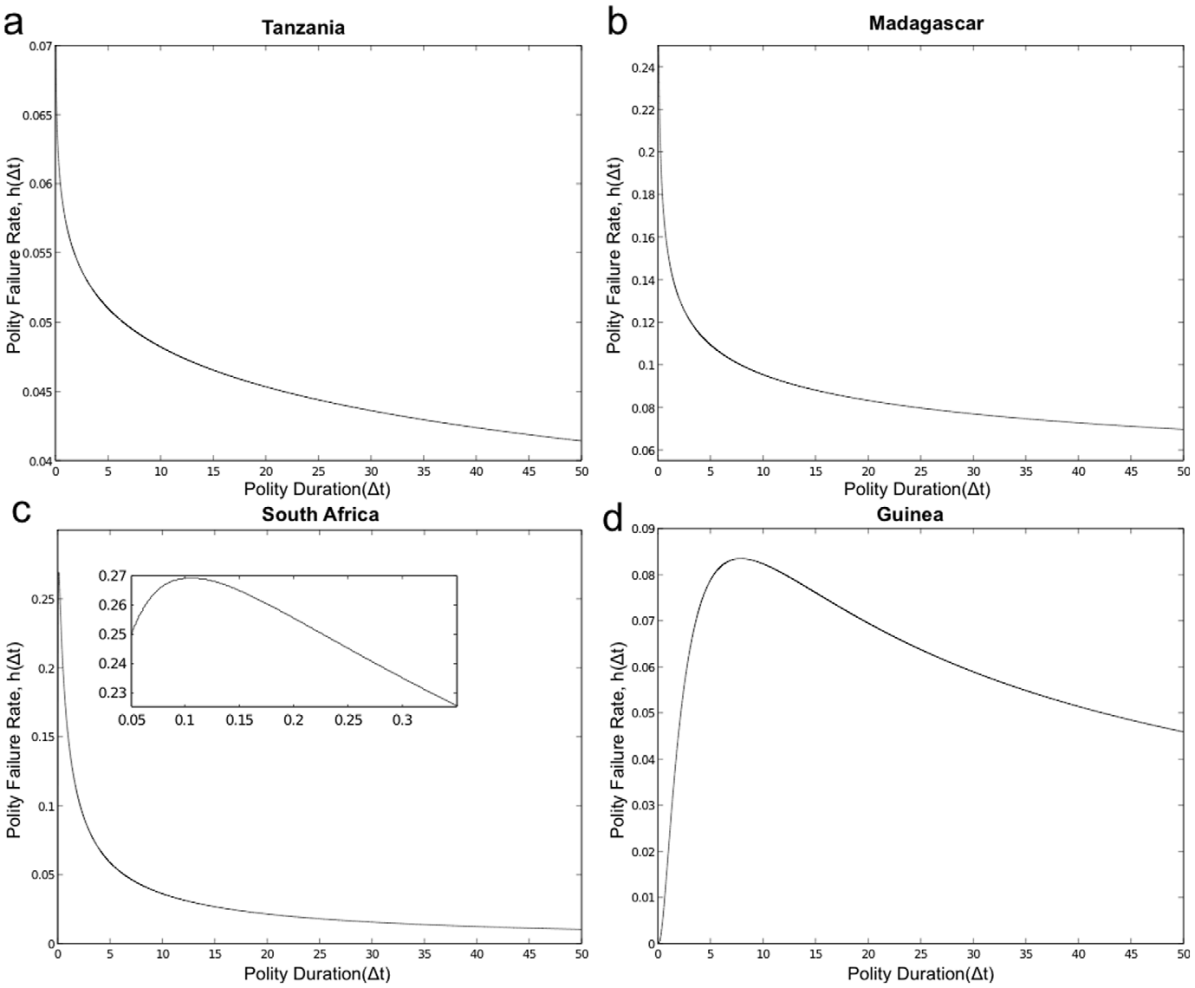
Polity Failure Rates. The figures show different properties for polity failure rates using estimated parameters and data sampled from eight different countries: (a) Tanzania with 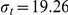, 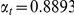, 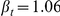; (b) Madagascar with 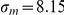, 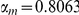, 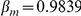; (c) South Africa with 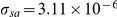, 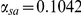, 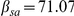; and (d) Guinea with 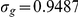, 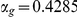, 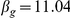, respectively. Madagascar and Tanzania exhibit monotonically decreasing, and South Africa and Guinea have uni-modal polity failure rates, respectively.

In contrast to U-shaped polity failure rate, we also observe that other countries exhibit different functional properties in which the risk of polity change initially starts low and increases to a maximum, before decreasing with uni-modal failure rate. Twenty-four countries have failure rates exhibiting uni-modal behaviors. Interestingly, seventy-five percent (75%) of the countries with this property have high to serious state fragility index. Monotonically increasing polity hazard has the largest number of countries with more than eighty-seven percents (87.50%) having high to serious state fragility index (e.g., see Fig. 2*c*). In contrast, countries with the monotonically decreasing countries are the most stable with low state fragility index. In general, monotonically increasing and decreasing polity failure rates have fewer countries with these risk structures. FigureS 3–4 illustrate common features in the structural property of polity failure rates for eight different countries, irrespectively of the polity score.

The picture that emerges from our socio-political reliability theory is that U-Shaped polity failure rate has both adaptation and damage cumulative phases. The size and the magnitude of each of these regions determine the nature of political instability. That is, two or more countries with similar features of polity failure rates can still have different course of socio-political instability depending on either the magnitude of the risk (see Figs. 4*a–b*) and/or the size of certain region (e.g., longer or shorter constant failure region; see Figs. 3*a–b*) in the case of bathtub shaped, the distance between the peak of uni-modal failure rate and the y-axis (see Figs. 4*c–d*), convexity and concavity of monotonically increasing and decreasing hazard rates (see Figs. 3*c–d*). For instance, Angola, Burkina Faso, Chad (e.g., see Fig. 3*a*), Comoros, Djibouti, Equatorial Guinea, Gabon, Gambia (e.g., see Fig. 3*b*), Mauritania, Morocco, Mozambique, and Sierra Leone are classified as having *U*-shaped risks of polity change. However, the socio-political environments such level of political factionalism, economic conditions, and structural details are different. Countries such as Burkina Faso, Comoros, Djibouti, Gabon, Gambia, Morocco, and Mozambique, for example, have shorter left tail (decreasing failure rate) followed by a longer constant failure than other countries within this cliometric space of shape parameters (see Figs. 3*a–b*). These properties consistently capture most of the political behaviors in many of these countries. Gambia with longer constant polity failure rate, for example, had a democratic polity that lasted for forty years before becoming an autocracy in recent years. Burkina Faso and Djibouti on the other hand have started to carefully liberalize their regimes. Other features of polity failure rates exhibiting similar socio-political interpretations were supported by microscopic processes. For instance, the socio-political conditions in Zimbabwe (e.g., see Fig. 3*d*) continue to deteriorate as illustrated by monotonically increasing polity failure rates and as a result the risk of polity change continues to increase. On the other hand, because of the longer constant rate region in the case of Libya, the socio-political condition is relatively stable as compared to Zimbabwe (see Figs. 3*c–d*). In contrast, we observed that countries with monotonically decreasing rates have lower rate of political instability. Countries that exhibit this property tend to be politically stable. For example, the political conditions in Madagascar and Tanzania have been relatively stable as captured by the monotone decrease in the polity failure rate (e.g., see Figs. 4*a–b*). Interestingly, South Africa has an uni-modal polity failure rate. However, its functional signature is closely similar to that of monotonically decreasing failure rate with a steep initial increase. In the period leading to the unbanning of the ANC, the release of Nelson Mandela, and the repeal of apartheid policy, there was heightening risk of possible political unrests and changes leading to policies similar to the redistributive policies that were adopted by Zimbabwe after its independence. However, South Africa has been relatively stable since the apartheid era.

### Conclusion

In conclusion, we find that the shift away from autocratic forms of governance in Africa provides strong evidence of the relationships between political violence and instability and democratization; and structural properties of the polity failure rates provide a mean to characterizing the risk of political instability in African countries, and other developing countries. Future research could illustrate whether greater understanding of the instantaneous behaviors of polity change coupled with regime type and other socio-political and economic factors can be used to design preventive and effective policies and/or mitigate political instability. An extension of our model using repairable system reliability theory with decision models may allow us to develop effective policies that minimize the cost of de-consolidating political institutions and the heightening risks of state failure, civil conflict and unrest.

## Supporting Information

Appendix S1 (PDF)

## References

[1] Auvinen J (1997). Political conflict in less developed countries 1981-89 (vol 34,
pg 177, 1997).. Journal of Peace Research.

[2] Bratton M, Van de Walle N (1997). Democratic experiments in Africa: regime transitions in
comparative perspective..

[3] Casper G, Taylor-Robinson MM (1996). Negotiating democracy: transitions from authoritarian rule. ,.

[4] Jaggers K (1995). Tracking democracy's third wave with the polity iii
data.. Journal of Peace Research.

[5] Collier P, Hoeffler A (1998). On economic causes of civil war.. Oxford Economic Papers.

[6] Huntington S (1991). The third wave: democratization in the late twentieth
century..

[7] Ellingsen TJ (2000). Colorful community or ethnic witches' brew?. Journal of Conflict Resolution.

[8] Ellingsen TNPG (1997). In Causes of Conflict in Third World Countries, Oslo: North/South
Coalition and International Peace Research Institute (PRIO)., chapter
Democracy and Armed Conflict in the Third World..

[9] Francisco RA (1995). The relationship between coercion and protest.. Journal of Conflict Resolution.

[10] Gurr TR (1974). Persistence and change in political systems,
1800-1971.. American Political Science Review (1927).

[11] Gurr TR (1993). Why minorities rebel: A global analysis of communal mobilization
and conflict since 1945.. International Political Science Review.

[12] Ellingsen T, Hauge W (1998). Beyond environmental scarcity: Causal pathways to
conflict.. Journal of Peace Research.

[13] Heldt B (1997). The dependent variable of the domestic-external conflict
relationship: Anecdotes, theories and systematic studies.. Journal of Peace Research.

[14] Hibbs DA (1973). Mass Political Violence: A Cross-National Causal
Analysis.. Wiley.

[15] Horowitz DL (1993). Democracy in divided societies.. Journal of Democracy.

[16] Cliophysics (e.g., Cliodynamics). applies the theories and formal mathematical methods to study historical processes, e.g., dynamics and statistical regularities.

[17] Lichbach MI (1987). Deterrence or Escalation?. Journal of Conflict Resolution.

[18] Moore W (1998). Repression and dissents substitution, context, and
timing.. American Journal of Political Science.

[19] Lichbach MI (1995). The rebel's dilemma..

[20] Vanhanen T (1999). Domestic Ethnic Conflict and Ethnic Nepotism: A Comparative
Analysis.. Journal of Peace Research.

[21] Weede E, Muller EN (1990). Cross-national variation in political violence.. Journal of Conflict Resolution.

[22] Marshall MG (2010). Polity iv project: Political regime characteristics and
transitions, 1800–2071.. http://www.systemicpeace.org/polity/polity4.htm.

[23] Ted Robert GurrKJ, Moore WH (1989). Polity II Codebook..

[24] Krain M, Myers ME (1997). Democracy and civil war: A note on the democratic peace
proposition.. International Interactions.

[25] Levy J (1989).

[26] Vanhanen T (2000). A new dataset for measuring democracy, 1810-1998.. Journal of Peace Research.

[27] Ward M, Gleditsch K (1998). The american political science review; democratizing for
peace.

[28] Zanger SC (2000). A Global Analysis of the Effect of Political Regime Changes on
Life Integrity Violations, 1977-93.. Journal of Peace Research.

[29] Cherif A (2008). Sociopolitical Bundle and Reliability Theories under Nonlinear
Political Interactions: On The Sociopolitical Instability and Poitical
Dynamics of African States from 1946-2004..

[30] Collett D (1994). Modelling survival data in medical research..

