# Supplementary material for: Cliophysics: Socio-Political Reliability Theory, Polity Duration and African Political (In)stabilities
Source: PLoS One. 2010 Dec 29;5(12):e15169. doi: 10.1371/journal.pone.0015169 (PMC3012063; doi:10.1371/journal.pone.0015169)
Supplement: Appendix S1 — (PDF) [file pone.0015169.s001.pdf]

Appendix

In this section, we briefly outline the assumptions made to arrive at the equation in the paper. Since the properties of socio-political processes are highly non-intrinsic, non-self-averaging and dependent on extreme statistics, the use of extreme value distribution is therefore appropriate.

Motivated by the success of Weibull distribution and its various functional modifications in bio-demography and in the analyses of different types of lifetime dataset and processes, we use a more general distribution function for the lifetime of a polity subjected to a polity history $\sigma(\Delta t)$. The ansatz is given as follows:

$$\mathcal{F}(\Delta t; \sigma, \alpha, \beta) = \left(1 - \exp\left[-\Psi\left(\int_0^{\Delta t} \nu\left[\sigma(s)\right] ds\right)\right]\right)^{\beta} \tag{1}$$

where $\Psi(x)$ is the shape function, $\nu(\sigma)$ is the breakdown rule that governs the risk of polity change. This formalism allows us to obtain different distributional types (e.g., Weibull, exponential, Gompertz-Makeham and in their exponentiated forms for $\beta \neq 1$) for the lifetime distribution of polity under different parametric conditions.

The most widely use shape function in literature has favored a Weibull shape function of the form:

$$\Psi(x) = x^{\alpha} \tag{2}$$

with $\alpha = 1$ reducing to the exponential shape function. The use of power-law like shape function has its support in empirical and theoretical works in the study of many sociological and historical processes and reliability engineering (see [**?**] and references therein). In our paper, we assume a linear breakdown rule for the shake of simplicity and of minimal of use of parameters. However, one can use different breakdown rule yielding different distributional properties. For example, the two widely used special forms of breakdown rule that have been widely used in the literature are power-law and exponential breakdown rules. The power-law breakdown rule is scale-invariant and can be seen as a local approximation of the exponential breakdown rule, which has a characteristic failure rate.

The power-law has the form:

$$\nu(\sigma) = \nu_o \left(\frac{\sigma(t)}{\sigma_o}\right)^{\rho} \tag{3}$$

and the exponential breakdown rule is of the form:

$$\nu(\sigma) = \nu_o \exp\left(\frac{\sigma(t)}{\sigma_o}\right) \tag{4}$$

where ν, σ_o, and ρ are positive constants. Note that if we add a constant term in the exponential breakdown rule, e.g.: $\nu(\sigma) = \nu_o \left(\exp\left(\frac{\sigma t}{\sigma_o}\right) + c\right)$, and assume linear shape function Ψ and polity history σ with $\alpha = \beta = 1$, we recover Gompertz-Makeham law. This shows the flexibility of our formulation.

In order to obtain Eq. 1, we use the power-law breakdown rule with $\rho = 1$, and a constant polity history $\sigma(\Delta t)$. Without loss of generality, we chose $\nu_o = \sigma(\Delta t) = 1$. By substituting Eqs. 2-4 into Eq. 1, we obtain the exponentiated Weibull distribution in the paper. Letting the age or duration of polity, $\Delta t \geq 0$ be non-negative random variable, we denote the cumulative distribution $\mathcal{F}(\Delta t)$ by Eq. 1, density function by $\mathcal{F}'(\Delta t)$, and survivability function by $\mathcal{S} = 1 - \mathcal{F}$. Then the hazard or risk function of polity change is defined as $\mathcal{H} = -\frac{\mathcal{S}'}{\mathcal{S}} = \frac{\mathcal{F}'}{1-\mathcal{F}}$. The properties of the hazard function in the parametric space of α and β in order to characterize the four distinct regions of the risk of polity change are shown in Fig. 2a. We then fitted the distribution (e.g., Eq. 1) with all the assumptions above to individual polity durations for each countries. Here we list our estimation results for each country, where *NA* indicates that there is not enough data to obtain those statistical quantities.

Country	σ	α	β	SSE	R^2	RMSE	R^2_{adj}	DFE
Algeria	0.132	0.4564	100	0.06440424	0.864991111	0.126889953	0.831238889	4
Angola	9.92	1.353	0.563	0.026595971	0.935789726	0.094155848	0.892982877	3
Benin	0.00616	0.2493	100	0.039817994	0.940107101	0.075420719	0.931550972	7
Botswana	0.2916	0.4216	100	NA	NA	NA	NA	1
Burkina Faso	10.53	8	0.07983	0.029993641	0.967227228	0.057728908	0.95994439	9
Burundi	2.531	1.433	1.731	0.064972511	0.929007309	0.084965686	0.913231155	9
Cameroon	0.3171	0.5035	100	0.023417469	0.94346354	0.076513836	0.929329425	4
Central African Republic	0.0002487	0.1617	100	0.030407613	0.908016971	0.100677063	0.877355962	3
Chad	14.81	1.946	0.2602	0.0579623	0.912815041	0.098287249	0.883753388	6
Comoros	4.991	4.039	0.198	0.082015672	0.924203099	0.08634796	0.910421844	11
Congo Brazzaville	5.994	0.9218	1.172	0.023296881	0.959923818	0.068259623	0.943893345	5
Congo Kinshasa	0.0009598	0.1948	100	0.157812775	0.682965407	0.177658535	0.619558488	5
Djibouti	20.02	11.68	0.06025	0.03189426	0.870828245	0.178589643	0.612484735	1
Egypt	0.0003892	0.1625	84.91	0.021445241	0.963109079	0.065490825	0.948352711	5
Equatorial Guinea	38.01	5.419	0.08149	0.006372967	0.974189485	0.079830862	0.922568456	1
Ethiopia	0.001112	0.1869	99.97	0.016871618	0.979715714	0.04592333	0.974644642	8
Gabon	34.33	7.678	0.07926	0.015670581	0.936534146	0.125182192	0.809602438	1
Gambia	20.89	9.487	0.06074	0.020204526	0.951220502	0.082066083	0.918700837	3
Ghana	3.61	1.451	1.104	0.065395033	0.939563492	0.077103845	0.928575036	11
Guinea	0.9492	0.4285	11.03	2.30E-04	0.999067917	0.015170493	0.997203752	1
Guinea Bissau	2.297	0.9065	2.27	0.030201545	0.954571842	0.070947804	0.939429123	6
Ivory Coast	0.002964	0.2277	100	0.044597764	0.910406278	0.094443384	0.892487533	5
Kenya	6.423	1.431	0.729	0.027550557	0.963182438	0.062735906	0.952663134	7
Lesotho	0.2442	0.4439	19.42	0.037388585	0.943761337	0.078939412	0.925015116	6

Country	σ	α	β	SSE	R^2	RMSE	R^2_{adj}	DFE
Liberia	0.0003443	0.1663	100	0.040573538	0.955667026	0.063697361	0.951233728	10
Libya	26.43	14.96	0.07934	0.00802356	0.967504583	0.089574325	0.902513749	1
Madagascar	7.698	0.7777	1.044	0.025084525	0.949606982	0.079190474	0.924410472	4
Malawi	0.0001574	0.1658	99.99	0.042631118	0.91435713	0.103236522	0.871535694	4
Mali	11.48	4.474	0.3056	0.017483771	0.964876353	0.066113106	0.947314529	4
Mauritania	48.31	4.768	0.04607	0.097604444	0.803919644	0.156208549	0.705879467	4
Morocco	17.74	8.557	0.08836	0.029081167	0.941578012	0.085266006	0.912367018	4
Mozambique	18.18	13.96	0.0639	0.02725606	0.889612956	0.165094095	0.668838868	1
Niger	4.665	1.4	1.162	0.044620445	0.94037086	0.079839522	0.923333963	7
Congo Kinshasa	0.0009598	0.1948	100	0.157812775	0.682965407	0.177658535	0.619558488	5
Nigeria	1.079	0.5629	4.068	0.047935515	0.942368438	0.077407618	0.927960547	8
Rwanda	0.004701	0.2306	100	0.026503734	0.946755891	0.072806228	0.936107069	5
Senegal	0.9108	0.4164	4.778	0.016886308	0.966076614	0.064973663	0.949114921	4
SierraLeone	5.237	1.14	0.7098	0.143227187	0.856576072	0.119677562	0.827891286	10
Somalia	0.6683	0.5671	100	0.034823003	0.85896684	0.131952648	0.78845026	2
South Africa	3.36E-06	0.1046	69.96	0.002124603	0.991395357	0.04609342	0.97418607	1
Sudan	0.01355	0.2734	60.6	0.072715249	0.912576439	0.095338377	0.890720549	8
Swaziland	0.01881	0.2632	100	0.031698254	0.87162207	0.125893316	0.807433106	2
Tanzania	19.26	0.8893	1.06	NA	NA	NA	NA	0
Togo	16.03	14.71	0.07292	0.019616474	0.952640226	0.080863	0.921067044	3
Tunisia	11.51	9.089	0.1878	0.02691302	0.945933664	0.082025942	0.918900496	4
Uganda	1.42	0.6284	2.98	0.032610113	0.964368321	0.060194235	0.95645017	9
Zambia	0.2571	0.5457	100	0.050891428	0.897762756	0.10088749	0.877315307	5
Zimbabwe	7.15	1.133	0.8863	0.018194308	0.963448935	0.067443139	0.945173402	4
